# ZNF473 promotes colorectal cancer progression and chemoresistance by destabilizing p53 protein to upregulate Survivin

**DOI:** 10.1038/s41420-026-03145-4

**Published:** 2026-05-05

**Authors:** Yunhua Xu, Guang Fu, Qing Fang, Lan Liao, Xiangwen Tan, Xiong Li, Shuxiang Li, Kai Fu, Shuai Xiao

**Affiliations:** 1 Department of Pharmacology, School of Pharmacy, Tongji Medical College, Huazhong University of Science and Technology, Wuhan, China; 2 Department of Gastrointestinal Surgery, The First Affiliated Hospital, Hengyang Medical School, University of South China, Hengyang, China; 3 Department of Pathology, The First Affiliated Hospital, Hengyang Medical School, University of South China, Hengyang, China; 4 First Clinical Medical College, Lanzhou University, Lanzhou, China; 5 Institute of Molecular Precision Medicine, Hunan Key Laboratory of Molecular Precision Medicine, Xiangya Hospital, Central South University, Changsha, China; 6 Cancer Research Institute, The First Affiliated Hospital, Hengyang Medical School, University of South China, Hengyang, China

**Keywords:** Colorectal cancer, Cell growth, Chemotherapy

## Abstract

Colorectal cancer (CRC) is a leading cause of cancer-related mortality worldwide. The classic development of CRC is a process from normal colonic mucosa to polyp to eventually adenocarcinoma. However, the critical genes regulating this process and the underlying molecular mechanisms remain elusive. Here, we identified ZNF473 as an upregulated and key functional gene in CRC progression. Specifically, comprehensive bioinformatics analyses were performed to explore the expression of ZNF473 in CRC samples and to investigate its correlation with clinicopathological characteristics, prognosis, and potential biological functions. In vitro experiments were performed to elucidate the potential role and molecular mechanisms of ZNF473 in CRC progression. Results demonstrate that ZNF473 is highly expressed in CRC and correlates with poor prognosis. Functionally, ZNF473 knockdown significantly inhibits cell viability and proliferation. Furthermore, gene function enrichment analyses reveal an association between ZNF473 and pathways related to drug metabolism (Cytochrome P450) and chemotherapy resistance. Mechanistically, ZNF473 physically interact with p53 to promote its protein degradation, consequently upregulates the Survivin expression. In summary, this study reveals the role and molecular function of ZNF473 in CRC progression, uncovering a potential novel ZNF473/p53/Survivin axis and providing a hint for targeting ZNF473 to suppress tumor growth and potential chemoresistance.

## Introduction

Colorectal cancer (CRC) remains a major global health challenge, ranking among the leading causes of cancer-related morbidity and mortality worldwide [1, 2]. Recent study indicates that the United States accounts for approximately 154,270 new cases, and estimated 52,900 deaths annually [1]. Furthermore, the global incidence and mortality of CRC will dramatically increase to 3.2 million and 1.6 million by 2040, respectively, thus causing a heavy burden on healthcare systems and medical expenditures globally [3]. Although advancements in screening and treatment strategy have contributed to a gradual decline in the overall CRC morbidity and mortality over recent decades, the incidence has been steadily rising in younger populations [4]. Moreover, nearly 25% CRC cases exhibit distant metastasis, for which the 5-year survival rate remains alarmingly low at only 16.4%[5]. Given these challenges, a profound understanding of the molecular mechanisms underlying CRC initiation and progression is urgent for improving clinical outcomes. Since sustained cell proliferation is a fundamental hallmark of cancer, including CRC [6], elucidating the regulatory networks governing this process is crucial for identifying novel therapeutic targets and enhancing patient survival.

Zinc finger proteins (ZNFs) constitute a large family of transcription factors (TFs). The stable zinc finger structure allows ZNFs to recognize and bind to specific DNA and RNA sequences, enabling them to play crucial roles in diverse biological processes, such as transcriptional regulation, chromatin remodeling, protein/RNA homeostasis, and DNA repair [7, 8]. Recent studies have continuously highlighted the critical roles of ZNFs in human health and disease, and their dysregulations are frequently observed in various cancers [9, 10]. For example, ZNF322A enhances the stem cell-like features of lung cancer by directly binding to histones and transcriptionally repressing c-Myc expression [11]. Another study demonstrated that ZBTB20 promotes the proliferation and invasion of gastric cancer via the NFKBIA/NF-κB signaling pathway [12].

Zinc Finger Factor 473 (ZNF473), also known as ZFP100, is a distinct member of the ZNF family. Previous studies have linked it to diverse pathological conditions, including congenital lung malformations, osteoporosis risk, and sensitivity to 5-fluorouracil [13–15]. Notably, its association with chemotherapy sensitivity suggests a potential role in regulating cellular survival or drug response mechanisms in epithelial malignancies. Despite these findings, the specific biological function and underlying molecular mechanisms of ZNF473 in CRC remain largely unknown.

In this study, we explored the transcriptome profiles during the classic adenoma-carcinoma sequence of CRC and discovered that ZNF473 was significantly highly expressed in both polyps and tumors. We subsequently investigated the correlation between ZNF473 expression and CRC development and prognosis, and sought to delineate its molecular function and underlying mechanisms. Our findings demonstrate that ZNF473 is significantly overexpressed in CRC tissues and is associated with poor patient prognosis. Functionally, we revealed that ZNF473 promotes CRC cell viability and proliferation. Mechanistically, we discovered that ZNF473 directly interacts with the p53 protein, thereby potential negatively modulating the p53/Survivin signaling pathway. Collectively, our findings elucidate the oncogenic role of ZNF473 in CRC progression, offering a novel molecular foundation and identifying a potential therapeutic target for CRC management.

## Results

### ZNF473 is highly expressed in CRC

**Fig. 1:**
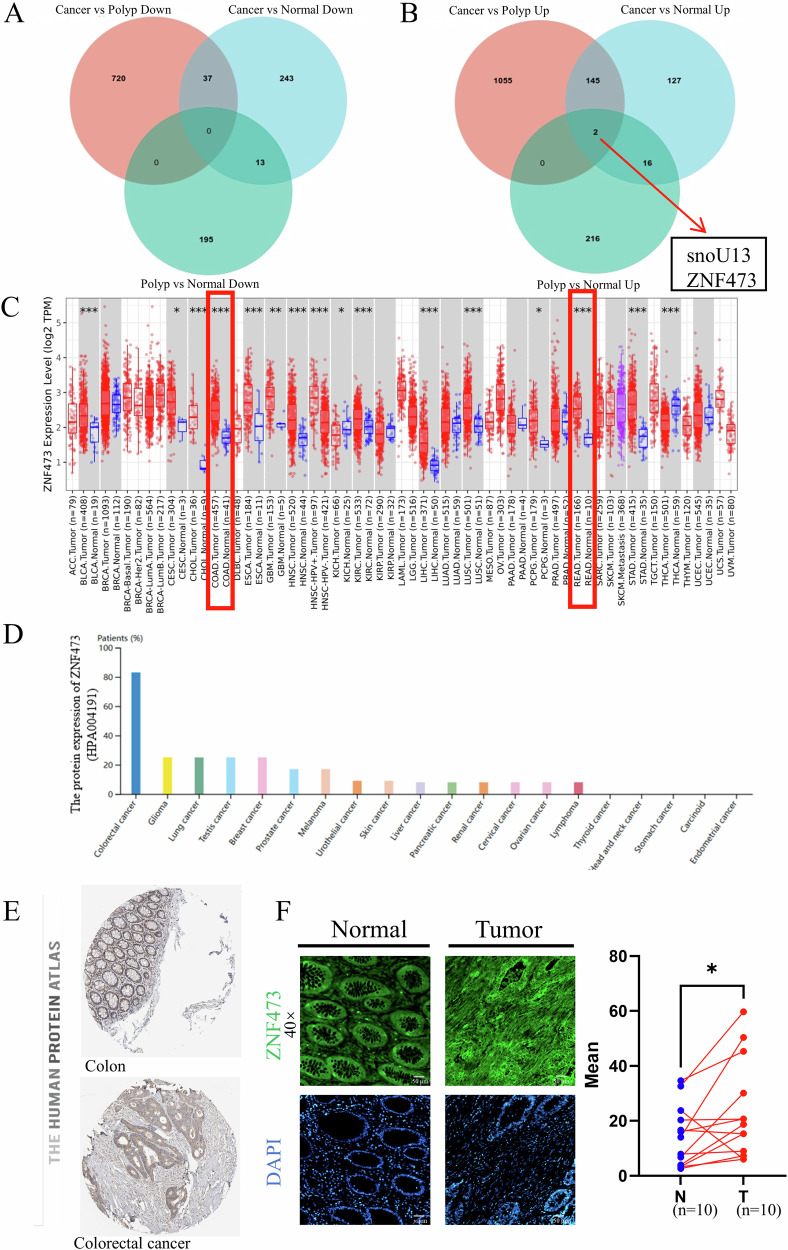
ZNF473 is significantly upregulated in colorectal cancer. **A, B** the Venn diagram of commonly down-regulated or up-regulated genes between cancer vs. polyp, cancer vs. normal, and polyp vs. normal, respectively. **C** the box plot of ZNF473 mRNA expression level in pan-cancer in the TCGA database. The reb box and blue box represent tumor and normal samples, respectively. **D** the protein expression level of ZNF473 in pan-cancer in the HPA database. **E** the representative immunohistochemical result of ZNF473 in CRC tumors and normal tissues in the HPA database. **F** representative image of IF results of ZNF473 in CRC clinical tumor and normal samples, the scale bar is 50 μm. The fluorescence intensity of ZNF473 across different samples was quantified using the mean fluorescence intensity. N Normal, T Tumor, vs. versus, *p < 0.05, **p < 0.01, ***p < 0.001.

To investigate the specific genetic alterations driving CRC progression, we performed RNA-seq on CRC specimens as our previous reported [16]. Comprehensive bioinformatic analysis was performed to compare gene expression profiles among normal mucosae, polyps, and adenocarcinomas, revealing distinct transcriptomic landscapes across these types. To identify genes consistently deregulated throughout CRC evolution, we further conducted Venn analysis. This analysis revealed 37 and 13 genes that were commonly downregulated in “cancer vs. polyp” and “cancer vs. normal” groups, and “cancer vs. normal” and “polyp vs. normal” groups, respectively; However, no downregulated genes were shared across all three comparison sets (Fig. 1A). Intriguingly, Zinc Finger Factor 473 (ZNF473) and Small nucleolar RNA U13 (snoU13) emerged as the only candidates who consistently upregulated across all comparisons (Fig. 1B). Given that only ZNF473 is protein-coding gene among these upregulated candidates, we supposed that it may play a pivotal role in the initiation and progression of CRC.

To validate the expression profile of ZNF473, we first explored its mRNA expression across various cancers using the TCGA database. The results indicated that ZNF473 is significantly highly expressed in multiple tumor types, including Colon Adenocarcinoma (COAD) and Rectal Adenocarcinoma (READ) (Fig. 1C). Furthermore, proteomic profiling across pan-cancer samples via the Human Protein Atlas (HPA) database also revealed that ZNF473 protein levels were notably higher in CRC than in other tumors (Fig. 1D). Consistent with these findings, immunohistochemical (IHC) staining results from HPA also indicated significantly elevated ZNF473 protein expression in CRC tumor tissue versus normal tissue (Fig. 1E).

To corroborate these public database findings within our clinical cases, we collected 10 paired samples of tumor and their matched adjacent normal tissues from CRC patients who underwent radical surgery. We assessed ZNF473 protein expression via immunofluorescence (IF) staining. The results showed that ZNF473 was upregulated in CRC tumor tissues compared to corresponding adjacent tissues, with prominent immunoreactivity observed in both the cytoplasm and the nucleus (Fig. 1F). Quantification of IF intensity further showed that ZNF473 expression was elevated in the majority of cancer tissues relative to para-cancerous tissues (Fig. 1F). Collectively, these results indicate that ZNF473 is overexpressed in CRC, further indicating its functional involvement in CRC progression.

### High ZNF473 expression level correlates with poor prognosis in CRC

**Fig. 2:**
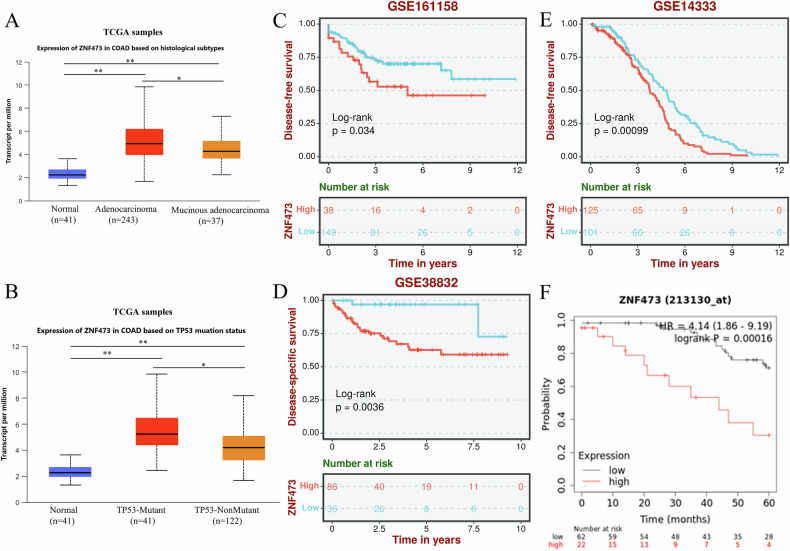
High ZNF473 expression is associated with poor prognosis in CRC patients. **A** The box plot of ZNF473 transcriptional level in different histological subtypes of TCGA-COAD samples. **B** The box plot of ZNF473 transcriptional level in different TP53 mutation statuses of TCGA-COAD samples. **C–E** the ‌Kaplan-Meier survival curve‌ of ZNF473 in GSE161158, GSE38832, and GSE14333 GEO datasets. F, the ‌Kaplan-Meier survival curve‌ of ZNF473 in ‌CMS2 (canonical) subtype in the TCGA database. *, *p* < 0.05, **, *p* < 0.01, ***, *p* < 0.001.

To further investigate the clinical significance of ZNF473 in CRC, we analyzed its correlation with clinicopathological features and prognosis of CRC patients using the UALCAN [17] and Gene Expression Omnibus (GEO) database. UALCAN-based analysis revealed that ZNF473 was significantly overexpressed across various histological subtypes of CRC (adenocarcinoma and mucinous adenocarcinoma) compared with normal colon tissues (Fig. 2A). Furthermore, we observed a striking association between ZNF473 expression and TP53 mutation status, with the highest ZNF473 expression levels detected in colon adenocarcinoma samples harboring TP53 mutations (Fig. 2B). Similar expression patterns regarding histological subtypes and TP53 status were also observed in rectal cancer (data not shown), which consistent with colon cancer.

Next, we explored the prognostic impact of ZNF473 expression using several independent GEO datasets that contain clinical survival information, specifically GSE38832 [18], GSE161158 [19], GSE14333 [20]. Kaplan-Meier analysis revealed that patients with high ZNF473 expression level exhibited significantly worse disease-free survival (DFS) compared to those in the low-expression group across all three cohorts (Fig. 2C–E). Subsequently, we explored patients who had not received chemotherapy and performed a comprehensive analysis of ZNF473 on overall survival (OS) stratified by Consensus Molecular Subtypes (CMS) classification based on TCGA. Results showed that ZNF473 had the most significant detrimental effect on the prognosis of the Classic type (CMS2) (Fig. 2F). Collectively, these findings suggest that ZNF473 expression varies according to CRC histological and molecular subtypes and TP53 mutation statuses, and crucially, its high expression is closely associated with poor prognosis in CRC.

### Knockdown of ZNF473 inhibits cell proliferation and viability in CRC

**Fig. 3:**
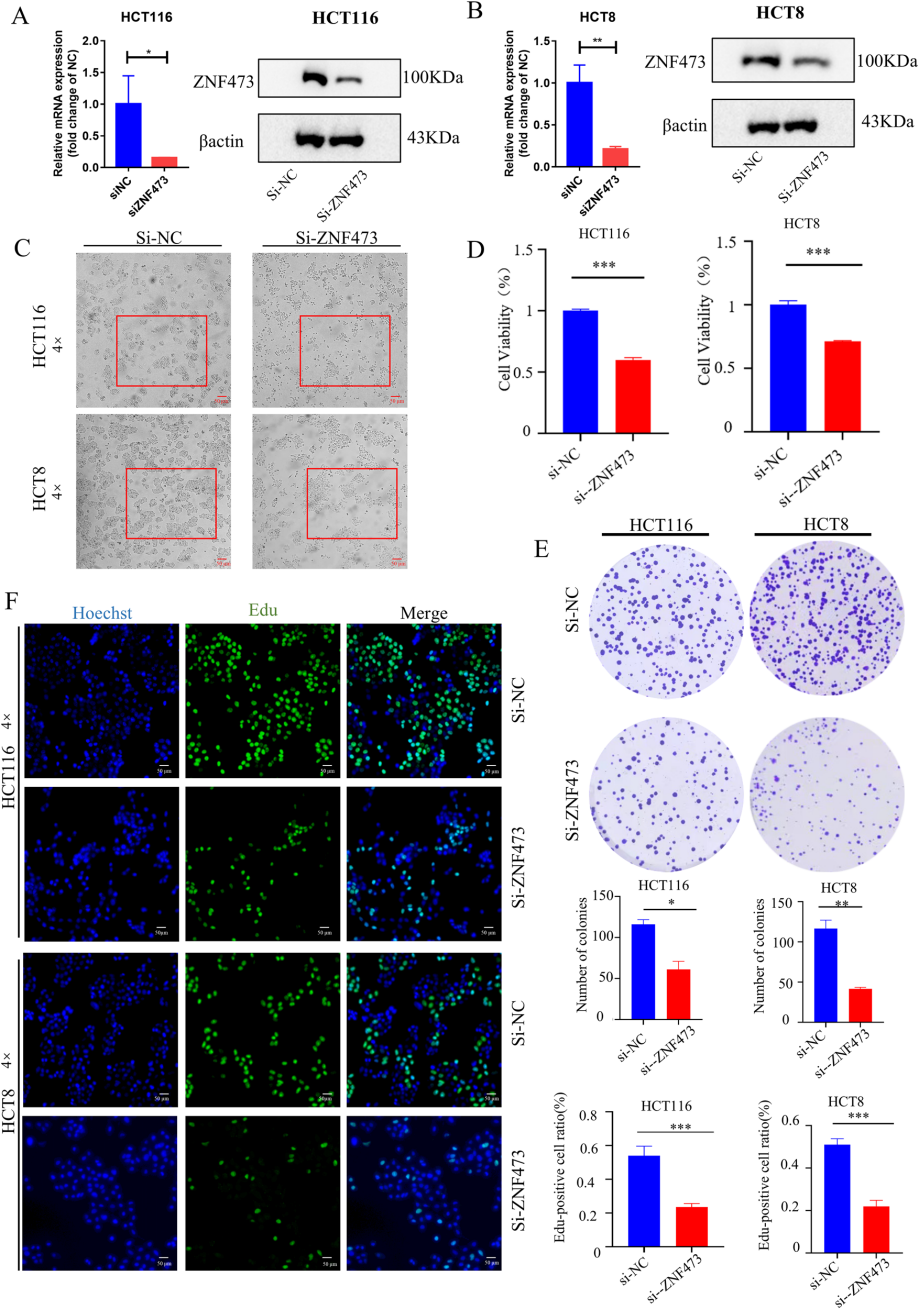
Knockdown of ZNF473 inhibits CRC cell proliferation and viability. **A, B** the efficiency of ZNF473 mRNA and protein knockdown in HCT116 and HCT8 cells. **C** representative images of cell viability observed under a light microscope after ZNF473 knockdown in HCT116 and HCT8 cells. **D** the bar chart of cell viability after ZNF473 knockdown for 48 h in HCT116 and HCT8 cells as detected by CCK-8 assay. **E** cell colony formation after ZNF473 knockdown in HCT116 and HCT8 cells. The colony number for each group is shown in a bar chart on the right. **F** the representative EdU fluorescence images of cells were detected after ZNF473 knockdown in HCT116 and HCT8 cells. The ratio of EdU-positive cells for each group is shown in a bar chart on the right. Data represent mean ± SD of three independent biological replicates, the scale bar is 50 μm, *, *p* < 0.05, **, *p* < 0.01, ***, *p* < 0.001.

To elucidate its biological function of ZNF473, we performed loss-of-function assays by knocking down its expression in two CRC cell lines, HCT116 and HCT8, using small interfering RNA (siRNA). The knockdown efficiency was validated both at the mRNA and protein levels (Fig. 3A, B). Cellular morphology observation showed a marked decrease in cell density and altered cellular appearance in the ZNF473-knockdown groups compared to the control (Fig. 3C). The CCK-8 assay showed that cell viability was significantly impaired in both cell lines with ZNF473 expression knockdown (Fig. 3D). Furthermore, colony formation assays showed the number of colonies in the ZNF473-knockdown group was significantly reduced compared to the control group (Fig. 3E), indicating that ZNF473 is essential for the clonogenic capacity of CRC cells. In addition, we assessed the effect of ZNF473 knockdown on cell proliferation using EdU incorporation assay. Results showed that the intensity of EdU green fluorescence was significantly attenuated, and the percentage of EdU-positive cells was markedly reduced in the ZNF473-knockdown group compared to the control group in both cell lines (Fig. 3F). Collectively, these findings indicate that ZNF473 is essential for the maintaining proliferation and viability of CRC cells.

### ZNF473 expression correlates with drug metabolism and chemotherapy resistance pathways in CRC

**Fig. 4:**
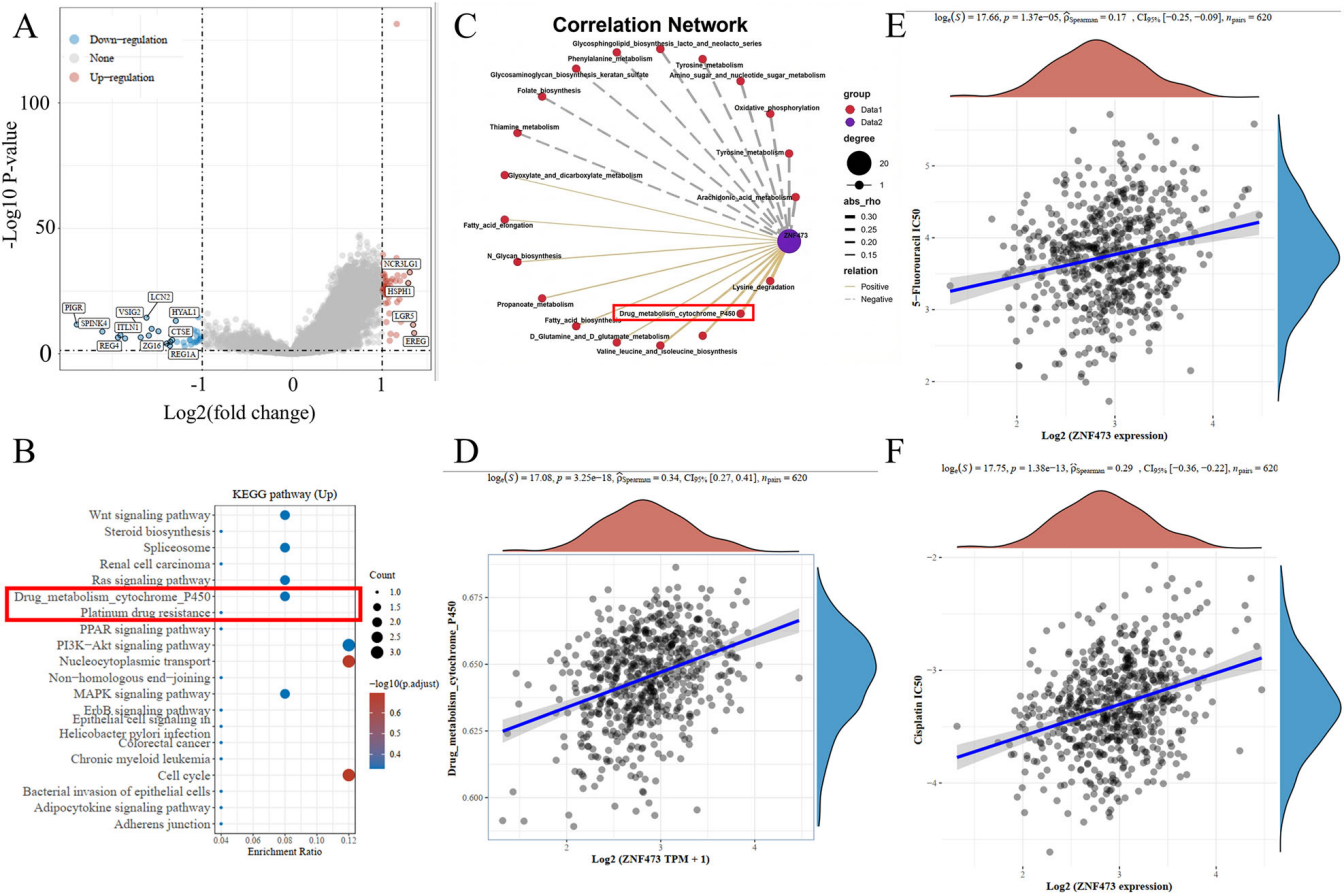
ZNF473 expression correlates with drug metabolism pathways and chemotherapy resistance. **A** the volcano diagram of differentially expressed genes in ZNF473 high and low expression groups. **B** the KEGG pathway enrichment analysis of up-regulated genes in the ZNF473 high-expression group. **C** the correlation network of ZNF473 expression and metabolic pathways. **D** the scatter plot of the correlation between ZNF473 expression and the Drug_metabolism_cytochrome_P450 pathway. **E, F** the scatter plot of the correlation between ZNF473 expression and the IC50 of 5-fluorouracil and cisplatin, respectively.

To elucidate the potential molecular mechanisms underlying the effect of ZNF473 on CRC cell proliferation and viability, we stratified TCGA-COREAD samples into high- and low-expression groups based on median ZNF473 expression levels and performed differentially expressed genes (DEGs) analysis (Fig. 4A). Subsequently, pathway enrichment analysis revealed that genes upregulated in the ZNF473-high group were significantly enriched in pathways related to drug metabolism and resistance, specifically “Drug_metabolism_cytochrome_P450” and “Platinum drug resistance”, suggesting a potential role for ZNF473 in chemotherapy resistance (Fig. 4B). Therefore, we analyzed the correlation between ZNF473 expression and various metabolic pathways within the TCGA dataset. The analysis identified a significant positive correlation between ZNF473 expression level and the “Drug_metabolism_cytochrome_P450” pathway signature (Fig. 4C, D). This further supports the speculation that ZNF473 is intimately involved in the metabolic processing of anti-tumor agents to effect chemoresistance. To validate this hypothesis, we explored the Genomics of Drug Sensitivity in Cancer (GDSC) database [21], which is the largest public pharmacogenomics repository (https://www.cancerrxgene.org/). Results revealed that ZNF473 expression was positively correlated with resistance to 5-Fluorouracil (5-FU) and Cisplatin (indicating higher IC50 values in cells with high ZNF473 expression) (Fig. 4E, F). Taken together, these data provide preliminary evidence that ZNF473 upregulation might contribute to chemotherapy resistance in CRC, potentially by modulating drug metabolism pathways.

### ZNF473 directly interacts with p53 and promotes its protein destabilization

**Fig. 5:**
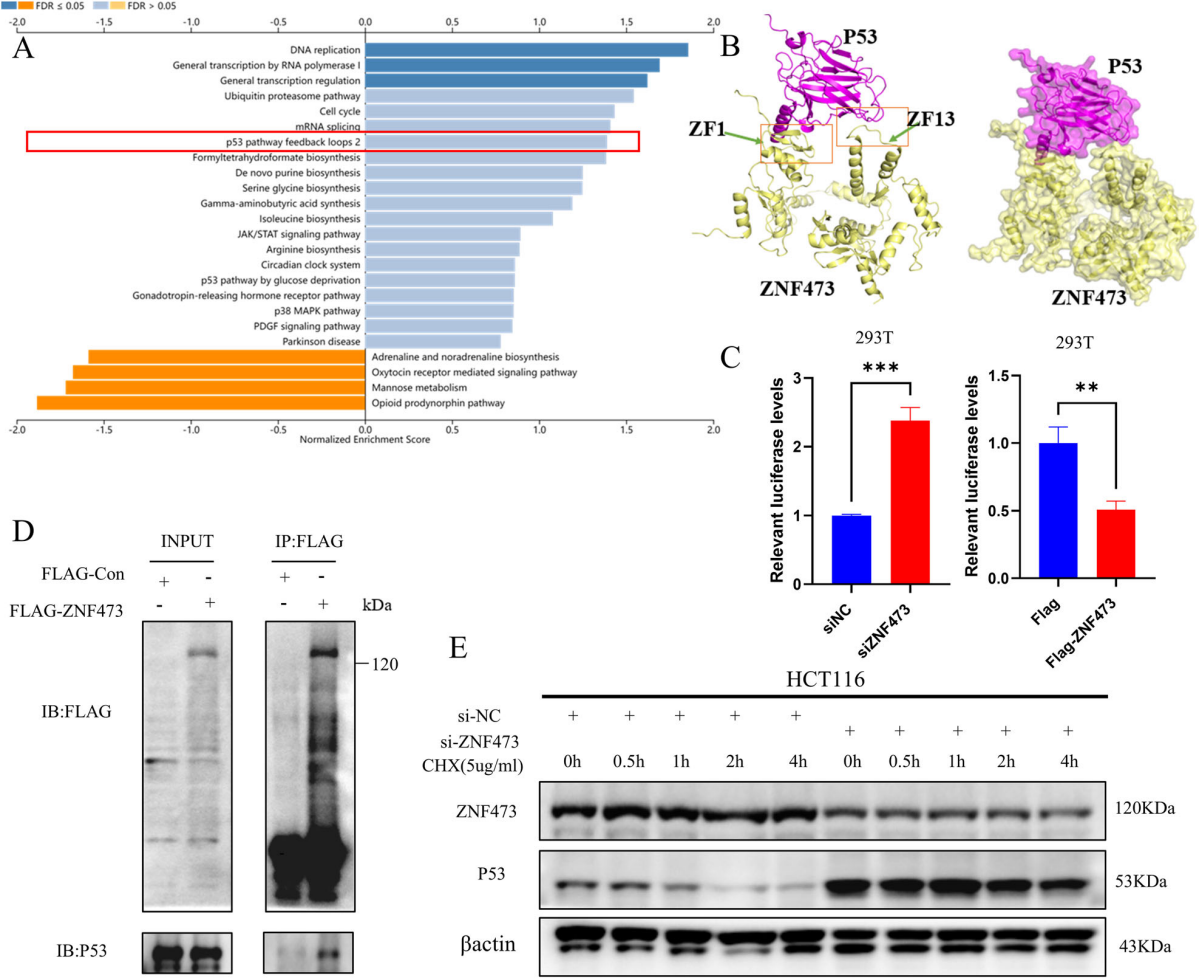
ZNF473 directly interacts with p53 promoting its protein destabilization. **A** KEGG enrichment analysis of ZNF473-related genes. **B** The predicted 3D structure diagram of the interaction between ZNF473 and P53 proteins. **C** The ductal luciferase reporter assays in 293 T cells. **D** Co-IP analysis showing the physical interaction between ZNF473 and P53. **E** The WB results showing the effect of ZNF473 knockdown on P53 protein stability when Cycloheximide (CHX) was added for different durations. Data represent mean ± SD of three independent biological replicates. *p < 0.05, **p < 0.01, ***p < 0.001.

To elucidate the probable downstream signaling pathways of ZNF473 in CRC, we performed KEGG pathway enrichment analysis. The results indicated that ZNF473 expression is significantly associated with DNA replication, cell cycle, and p53 signaling (Fig. 5A). Given the results of Fig. 2B and Fig. 5A, and that the p53 signaling pathway is a cornerstone driver of CRC tumorigenesis [22], we speculate that ZNF473 may modulate CRC cell survival via p53. Then, we analyzed the structural features of ZNF473 and p53 proteins using protein structural modeling, which predicted potential interaction interfaces between the ZF1/ZF13 domains of ZNF473 and p53 (Fig. 5B). Since ZNF473 is a transcription factor, we examined its impact on p53 transcriptional activity. Dual-luciferase reporter assay in HEK293T cells revealed that ZNF473 knockdown significantly enhanced p53 transcriptional activity, whereas ZNF473 overexpression suppressed its activity (Fig. 5C). In addition, to confirm the physical interaction suggested by the structural prediction, we performed Co-IP assays in HCT116 cells which transfected with Flag-tagged ZNF473. As expected, endogenous p53 protein was successfully pulled down by Flag-tagged ZNF473 (Fig. 5D). Furthermore, to determine whether this interaction affects p53 protein stability, we conducted a cycloheximide (CHX) chase assay. Notably, the degradation rate of p53 was notably retarded in the ZNF473-knockdown group compared to controls (Fig. 5E). Collectively, these results indicate that ZNF473 physically associates with p53 and promotes its protein destabilization.

### ZNF473 upregulates Survivin expression via the p53 signaling axis

**Fig. 6:**
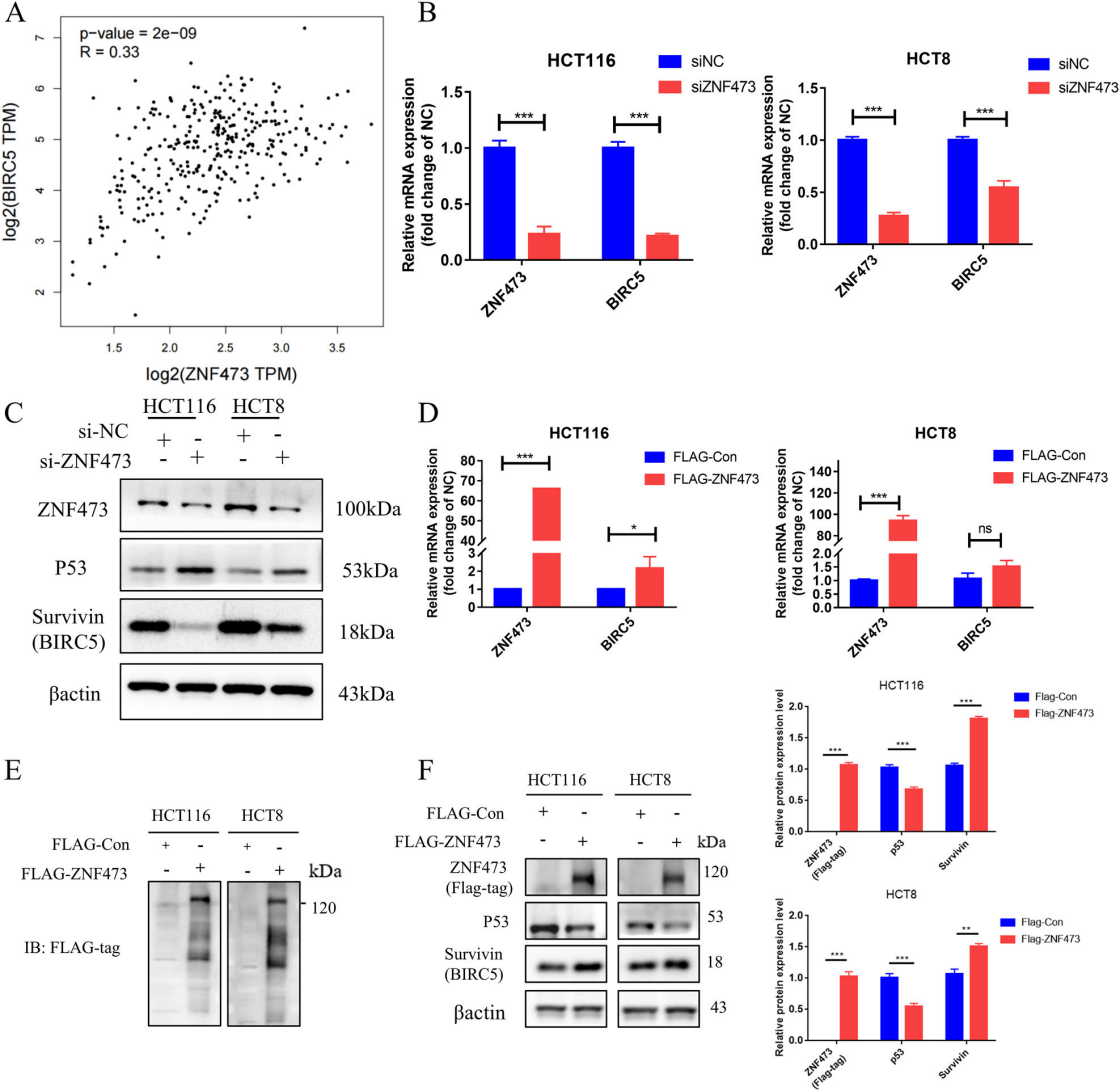
ZNF473 upregulates Survivin expression via the p53 signaling axis. **A** The scatter plot of the expression correlation between ZNF473 and BIRC5. **B–D** The effects of ZNF473 knockdown or overexpression on the mRNA and protein of P53 and BICR5 in HCT116 and HCT8 cells. **E** The protein overexpression efficiency of ZNF473 was detected by WB using a flag-tag antibody. **F** The effects of ZNF73 overexpression on P53 and Survivin proteins in HCT116 and HCT8 cells. Data represent mean ± SD of three independent biological replicates. *, *p* < 0.05, **, *p* < 0.01, ***, *p* < 0.001.

To further elucidate the probable molecular mechanism of ZNF473-mediated p53 regulation, we investigated the downstream targets of p53 in ZNF473 manipulated CRC cells. As p53 is a well-characterized transcriptional repressor of Survivin (encoded by BIRC5), a key anti-apoptotic protein, regulating its expression by binding directly to the BIRC5 promoter [21, 22]. Therefore, we explored the gene expression correlation between ZNF473 and Survivin protein coding gene BIRC5. Bioinformatic analysis revealed a significantly positively correlation between ZNF473 and BIRC5 expression (Fig. 6A). Then, we validated this regulation relationship in vitro. qRT-PCR results demonstrated that ZNF473 knockdown significantly reduced BIRC5 mRNA levels in both HCT116 and HCT8 cells (Fig. 6B). Consistent with these findings, WB results showed that ZNF473 knockdown led to a marked decrease in Survivin protein levels, accompanied by a reciprocal increase in p53 protein (Fig. 6C). Conversely, overexpression of ZNF473 upregulated BIRC5 mRNA (Fig. 6D). At the protein level, ZNF473 overexpression was verified by Co-IP using a Flag antibody (Fig. 6E), and we observed that it restored Survivin levels while suppressing p53 protein expression (Fig. 6F). Collectively, these findings reveal that ZNF473 physically interacts with and destabilizes p53 protein, thereby relieving its repression of Survivin, which suggest that ZNF473 possibly exerts its pro-tumorigenic role in CRC via the ZNF473/p53/Survivin axis.

## Discussion and conclusion

CRC remains a formidable global health challenge, characterized by persistently high incidence and mortality rates. Despite substantial progress in elucidating CRC pathogenesis, metastasis, and therapeutic strategies, the complex molecular networks are not yet fully demonstrated. The development of CRC typically recapitulates the classic adenoma-carcinoma sequence, a multi-step phenotypic transition from normal colonic mucosa to polyps, and ultimately to invasive adenocarcinoma. However, the pivotal genetic drivers governing these critical transitions remain largely elusive. In the present study we identified ZNF473 as the pivotal protein-coding gene consistently upregulated throughout the transitions from normal tissue to malignancy (Fig. 1A, B). This distinctive expression signature suggests that ZNF473 might play as a critical role in CRC evolution.

Zinc finger proteins (ZNFs) constitute the most extensive family of transcription factors in the human genome [23]. These proteins are pivotal orchestrators of malignant progression, primarily through their tight regulation of gene transcription and translation [10]. Growing evidence has implicated the dysregulation of various ZNF members in the pathogenesis of CRC. For instance, ZNF703 has been characterized as an oncogene that promotes CRC proliferation and metastasis [24]. Similarly, the ZNF746/c-Myc signaling play a critical role of Morusin-induced apoptosis in CRCs, which is mediated by the upstream miR193a-5p [25]. In contrast, ZNF277 was reported as a tumor suppressor, where it retards cell proliferation and senescence by inhibiting p21WAF1 expression [26]. Although ZNF473 has been previously linked to congenital lung malformations and 5-fluorouracil sensitivity [13–15], its specific function role and underlying mechanisms in tumorigenesis remained entirely unexplored. Here, we provide the first evidence that ZNF473 is significantly upregulated in CRC tissues and its overexpression servers as an indicator of poor prognosis (Figs. 1, 2).

To date, the molecular function of ZNF473 in tumorigenesis has remained largely unknown. Our study bridges this knowledge gap by establishing a direct functional link between ZNF473 dysregulation and CRC progression. We demonstrate that ZNF473 knockdown significantly impairs CRC cell proliferation and viability (Fig. 3). Beyond its role in driving tumor growth, bioinformatic and pharmacogenomic analyses also revealed a correlation between ZNF473 expression and chemotherapy resistance (e.g., to 5-FU and Cisplatin) and drug metabolism pathways (Fig. 4). These findings are coincident with previous report that ZNF473 expression is associated to 5-FU sensitivity in gastric cancer [15], suggesting that ZNF473 may not only prompt malignant progression but also provide a chemoresistant phenotype which is potentially mediated through the drug metabolism pathways or the activation of pro-tumor signaling cascades.

Mechanistically, our study elucidates a novel potential regulator axis of ZNF473/p53/Survivin in CRC progression. Gene enrichment analysis points to the p53 signaling pathway, a master regulator of cell cycle and apoptosis. We then discovered that ZNF473 directly interacted with the p53 protein, leading to its destabilization and subsequent degradation (Fig. 5). This finding is crucial because p53 is a cornerstone tumor suppressor which is essential for genomic integrity. Furthermore, we revealed a molecular link that p53 destabilizes its downstream target Survivin, a member of the inhibitor of apoptosis (IAP) family which have eight members in human, is a well-established canonical target repressed by p53 [27–30]. Numerous studies have shown that Survivin is closely involved in cell cycle progression [31], apoptosis inhibition [32], and cancer cell self-renewal [33] etc. Consequently, Survivin has been a focal point for targeted anti-cancer therapies for over two decades. Our data also identify that ZNF473 positively regulates Survivin expression (Fig. 6); specifically, the ZNF473-mediated p53 degradation relieves the repression of the Survivin, leading to Survivin expression upregulation. Therefore, we propose that ZNF473 exerts its pro-tumorigenic and potential chemoresistant effects through the ZNF473/p53/Survivin axis.

Nonetheless, there are still several limitations of the present study because of fund and experimental conditions restriction. First, we have extensively validated the function and mechanism roles of ZNF473 in vitro, but further in vivo experiments are needed. Second, although we demonstrate that ZNF473 promotes p53 destabilization, the precise biochemical pathway, specifically whether ZNF473 functions as a scaffold to recruit specific E3 ubiquitin ligases, remains to be fully elucidated. Third, given the striking correlation between ZNF473 and TP53 mutation status observed in clinical specimens, future studies using p53-mutant CRC cell lines to determine whether ZNF473 synergizes with gain-of-function p53 mutants are needed. Finally, direct experimental evidence using chemotherapy resistance models is needed to solidify the link between ZNF473 and chemoresistance. Therefore, our future studies will focus on addressing these limitations to fully elucidate the role of ZNF473 in CRC.

In conclusion, our study suggests ZNF473 as a pivotal oncogenic role in CRC. ZNF473 overexpression is closely associated with poor prognosis, and acts as an oncogene in promoting cell proliferation and survival, as well as potentially contributing to chemotherapy resistance in CRC. Mechanistically, ZNF473 directly interacts with the p53 protein, leading to its destabilization and subsequently relieving the repression of Survivin. Collectively, our findings elucidate the critical role of the ZNF473/p53/Survivin axis in CRC progression, offering a novel understanding CRC evolution and indicating ZNF473 as a potential therapeutic target for tumor growth and chemoresistance.

## Methods and materials

### Sample collection and RNA sequencing

A total of 50 surgical tissue specimens were collected for comprehensive transcriptome sequencing. The details regarding these samples and the sequencing procedure have been previously described in our report [16]. Briefly, the cohort consisted of 10 normal colonic mucosae, 10 polyps, 15 mucinous adenocarcinomas, and 15 adenocarcinomas. In addition, we additionally collected 10 paired samples of tumor and adjacent normal tissues from patients who underwent radical CRC surgery for experimental validation in this study.

Following total RNA extraction, cDNA libraries were generated via reverse transcription and subsequently sequenced. All experimental procedures were performed strictly according to the manufacturers’ protocols. The resulting RNA sequencing data were processed and subjected to comprehensive bioinformatic analysis as described in our previous study [16].

### Cell culture

The human CRC cell lines HCT116 and HCT8, and human embryonic kidney cells HEK293T were sourced from the American Type Culture Collection (ATCC). All cell lines were maintained in Dulbecco’s Modified Eagle Medium (DMEM, Gibco) supplemented with 10% fetal bovine serum (FBS, Gibco) and 1% penicillin-streptomycin (PS, Gibco). Cells were cultured in a humidified incubator at 37 °C with 5% CO_2_. The complete medium was refreshed every 2-3 days, and cells were routinely passaged using 0.25% trypsin-EDTA upon reaching 80–90% confluence.

### Immunofluorescence

Ten pairs of CRC tumor tissues and adjacent normal tissues were obtained from the Department of Pathology at our affiliated hospital. For IF staining, 5-μm-thick paraffin-embedded sections were deparaffinized, rehydrated, and subjected to heat-induced antigen retrieval in EDTA buffer (pH 9.0). After blocking with 5% bovine serum albumin (BSA) for 1 h at room temperature, sections were incubated overnight at 4 °C with the primary anti-ZNF473 antibody (1:100; YT6325, Immunoway). Following three washes with PBS, the sections were incubated with an Alexa Fluor 488-conjugated secondary antibody and counterstained with DAPI. Images were captured using a fluorescence microscope (D1, Zeiss).

### RNA interference

For ZNF473 knockdown, small interfering RNAs (siRNAs) targeting ZNF473 (si-ZNF473) and a non-targeting negative control siRNA (si-NC) were custom-synthesized by GenePharma (Suzhou, China). Cells were seeded into 6-well plates and transfected at 30–50% confluence using RNAiMAX (Invitrogen) according to the manufacturer’s instructions. The final concentration of siRNA used was 50 nM. Cells were harvested at 48 h post-transfection for subsequent knockdown efficiency verification and functional assays.

### ZNF473 over-expressed plasmid construction

The full-length human ZNF473 cDNA was amplified by PCR and subcloned into the pcDNA3.1-Flag-N expression vector. The recombinant plasmid (Flag-ZNF473) was validated by DNA sequencing. For overexpression experiments, cells were transfected 1 μg plasmid of either Flag-ZNF473 or the empty vector control (Flag-Con) using Lipofectamine 3000 (Invitrogen) according to standard protocols. Cells were harvested at 48 h post-transfection for efficiency expression validation and functional assays.

### Cell viability assay

Following transfection with si-ZNF473 or si-NC, HCT116 and HCT8 cells were seeded in 96-well plates at a density of 3,000 cells per well in 200 μL of culture medium. After 48 h of incubation, the supernatant was aspirated and replaced with 100 μL of CCK-8 working solution (prepared at a ratio of 1:10 between CCK-8 reagent and culture medium) per well. After an additional 4 h of incubation, the absorbance at 450 nm was measured. Cell viability was calculated and normalized to the si-NC group.

### RNA extract and Quantitative real-time PCR (qRT-PCR)

Total RNA was isolated from cultured HCT116 and HCT8 cells using TRIzol reagent (Takara) according to the manufacturer’s protocol. RNA concentration and purity were assessed using a NanoDrop 2000 spectrophotometer. For cDNA synthesis, 1 μg of total RNA was reverse-transcribed using the PrimeScript™ II High Fidelity RT-PCR Kit (Takara). Subsequently, qRT-PCR was performed suing SYBR Green qPCR Master Mix (Takara) on a LightCycler 480 System (Roche). Relative gene expression levels were calculated using the 2^^-△△Ct^ method and normalized to the internal control gene β-actin. The primer sequences used are:

β-actin-Forward primer: 5’-GATTCCTATGTGGGCGACGA-3’,

β-actin-Reverse primer: 5’-AGGTCTCAAACATGATCTGGGT-3’;

ZNF473-Forward primer: 5’-ATGAGGTGCCTCCACAACTTCC-3’,

ZNF473-Reverse primer: 5’-CCAGTTCCTTGGAGATGGTCTC-3’;

BIRC5-Forward primer: 5’- GATCTCTGAGCACATGCAGGTC-3’,

BIRC5-Reverse primer: 5’- GTTGGAGTCCTTGGTGACATTCC-3’.

### Western blotting

Total protein was extracted from cells or tissues using RIPA lysis buffer supplemented with protease and phosphatase inhibitor cocktails (Beyotime). Protein concentrations were determined using a BCA Protein Assay Kit (Beyotime). Equal amount of protein (20 μg per lane) was separated via 10% SDS-PAGE, and transferred onto PVDF membranes (Millipore). After blocking with 5% non-fat milk for 1 h at room temperature, the membranes were incubated overnight at 4°C with primary antibodies (all at 1:1000 dilution) against β-actin (51067-2-AP, Proteintech), ZNF473 (YT6325, Immunoway), Flag (20543-1-AP, Proteintech), P53 (10442-1-AP, Proteintech), and Survivin (10508-1-AP, Proteintech). Following three washes with TBST, the membranes were incubated with HRP-conjugated secondary antibodies for 1 h at room temperature. Protein bands were visualized using a high-sensitivity enhanced chemiluminescence (ECL) kit (KF8003, Affinity) and captured with the Bio-Rad ChemiDoc system.

### Co-immunoprecipitation (Co-IP)

For Co-IP analysis, HCT116 cells transfected with Flag-ZNF473 or empty vector (Flag-Con) were harvested and lysed in ice-cold IP Lysis Buffer (PR20037, Proteintech) supplemented with a protease and phosphatase inhibitor cocktail (Beyotime). The lysates were centrifuged at 12,000 rpm for 15 min at 4 °C, and the supernatants were collected. A portion of each supernatant was aliquoted as the “Input” control. The remaining lysates were incubated with an anti-Flag antibody (1:1,000, Proteintech) overnight at 4 °C with gentle rotation, followed by a 4-h incubation with Protein A/G magnetic beads (Thermo). Immunoprecipitates were washed three times with cold IP lysis buffer to minimize non-specific binding. Bound proteins were eluted by boiling in 2× SDS loading buffer for 10 min and subsequently analyzed by Western blotting using anti-Flag and anti-P53 antibodies to detect the bait and prey proteins, respectively.

### Cycloheximide chase assay

To assess the protein stability of p53, HCT116 cells were transfected with si-ZNF473 or si-NC. After 48 h of transfection, cells were treated with Cycloheximide (CHX) (Sigma-Aldrich) at a final concentration of 5 ug/mL to inhibit de novo protein synthesis. Cells were harvested at the indicated time points (0, 0.5, 1, 2, and 4 h) following CHX administration. The protein levels of p53 were subsequently determined by Western blotting, and its half-life was evaluated.

### Dual-luciferase reporter gene assay

HEK293T cells were seeded into 24-well plates and grown to 60–70% confluence. The cells were co-transfected with the LUC-P53 firefly luciferase reporter plasmid, the pRL-TK Renilla luciferase vector (as an internal control), and the indicated plasmids (Flag-ZNF473 or Flag-Con) or siRNAs (si-ZNF473 or si-NC) using Lipofectamine 3000. After 48 h of transfection, cells were lysed, and luciferase activities were measured using the Dual-Luciferase Reporter Assay System (Promega, USA) on a GloMax 20/20 luminometer according to the manufacturer’s protocol. The relative luciferase activity was calculated by normalizing the firefly luciferase signal to that of Renilla luciferase.

### Colony formation assay and EdU assay

For the colony formation assay, HCT116 and HCT8 cells transfected with si-ZNF473 or si-NC were seeded in 6-well plates at a density of 500–1000 cells per well. Cells were cultured for 10–14 days, with the medium replenished every 3 days. Once visible colonies had formed, they were fixed with 4% paraformaldehyde, stained with 0.1% crystal violet solution, and counted visually.

Cell proliferation was measured using an EdU (5-ethynyl-2’-deoxyuridine) incorporation assay kit (Vazyme). Cells were incubated with EdU labeling medium for 2 h, followed by fixation and permeabilization. EdU-positive cells were detected via the Click-iT reaction according to the manufacturer’s instructions. Nuclei were counterstained with DAPI. The percentage of EdU-positive (proliferating) cells was quantified under a fluorescence microscope (Zeiss).

### Difference expressed genes (DEGs) analysis

TCGA-CRC samples were stratified into high and low expression groups based on the median expression level of ZNF473. Differentially expressed genes (DEGs) between these groups were identified using the Limma R package (version 4.4.3), as previously described [34]. Specifically, the gene expression matrix was imported into R environment, the lmFit function was utilized for multiple linear regression, followed by the eBayes function to compute moderated t-statistics and log-odds of differential expression. The Benjamini-Hochberg (BH) method was used to adjust the p-value for multiple testing corrections. Genes with *p*-value ≤ 0.05 and a |log_2_ fold change | ≥1 are identified as DEGs.

### Gene function enrichment analysis

To investigate the functional implications of the identified DEGs, Kyoto Encyclopedia of Genes and Genomes (KEGG) pathway enrichment analysis was performed using the clusterProfiler R package (version 4.0). The DEGs were mapped against the whole-genome background to identify significantly enriched biological pathways. All parameters were kept at their default values. Pathways with *p*-value < 0.05 were considered to be significantly enriched. The code for comprehensive bioinformatics can be obtained by contacting the corresponding author with a reasonable request.

### Statistics

All data are presented as the mean ± standard deviation (SD) from at least three independent biological replicates. Statistical analyses were conducted using GraphPad Prism 7 (San Diego, USA) or R software. For comparisons between two groups, an unpaired, two-tailed Student’s t-tests, was used, while a paired t-test was applied for paired clinical samples. Differences among multiple groups were evaluated using one-way ANOVA followed by Tukey’s post-hoc test. For TCGA data, survival curves were generated using the Kaplan-Meier method and compared via the log-rank test. A *p*-value < 0.05 was considered statistically significant. Statistical significance is indicated as follows: *P < 0.05, **P < 0.01, ***P < 0.001.

## Supplementary information

Supplementary material

## Data Availability

The TCGA-COAD and TCGA-READ transcriptome data can be obtained from the TCGA public database. RNA-seq data can be obtained from the corresponding author upon reasonable request.
