# Supplementary figures and images for: ZNF473 promotes colorectal cancer progression and chemoresistance by destabilizing p53 protein to upregulate Survivin
Source: Cell Death Discov. 2026 May 5;12:277. doi: 10.1038/s41420-026-03145-4 (PMC13287752; doi:10.1038/s41420-026-03145-4)

**Figure 3A-B**

**
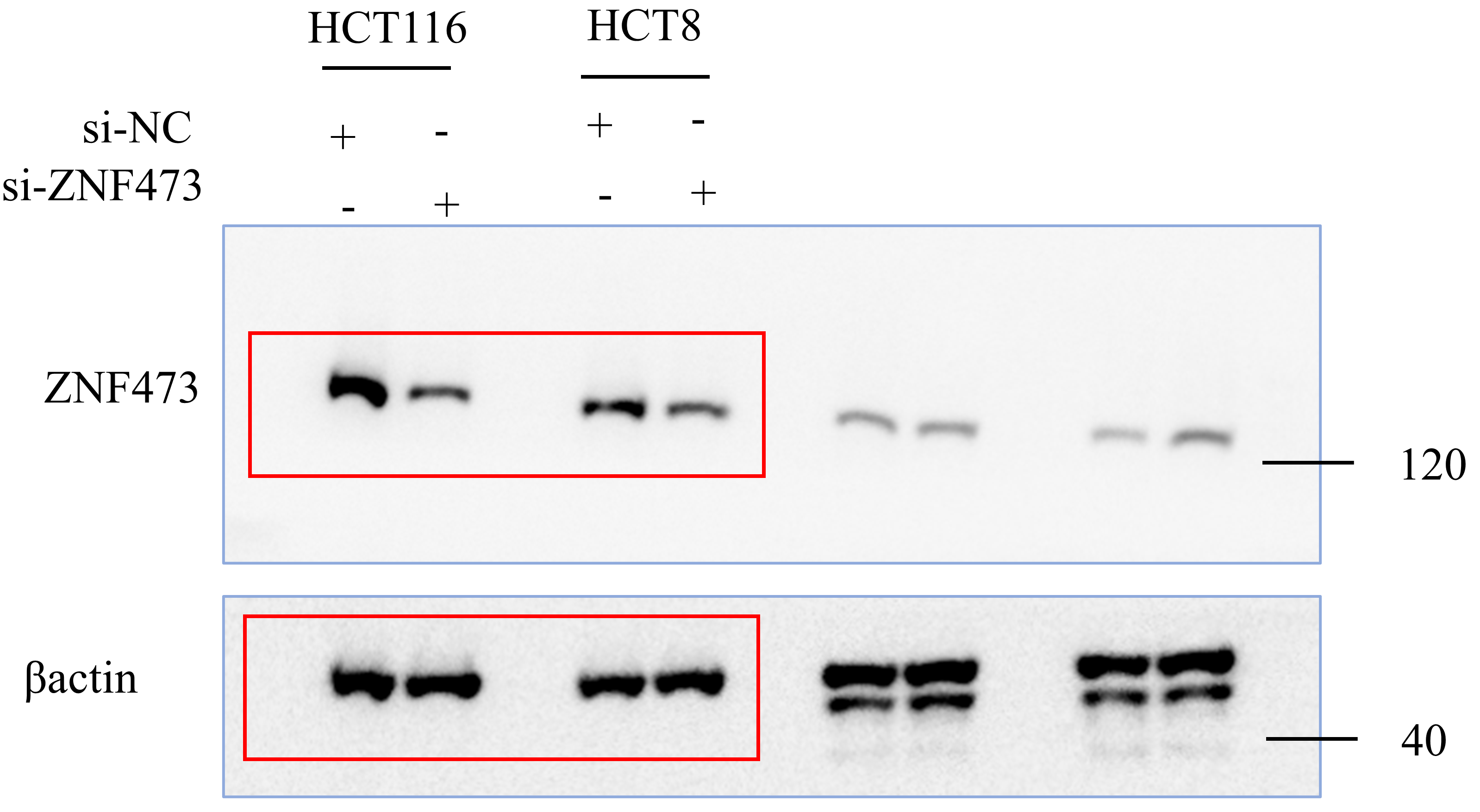
**

**Figure 5D**

**
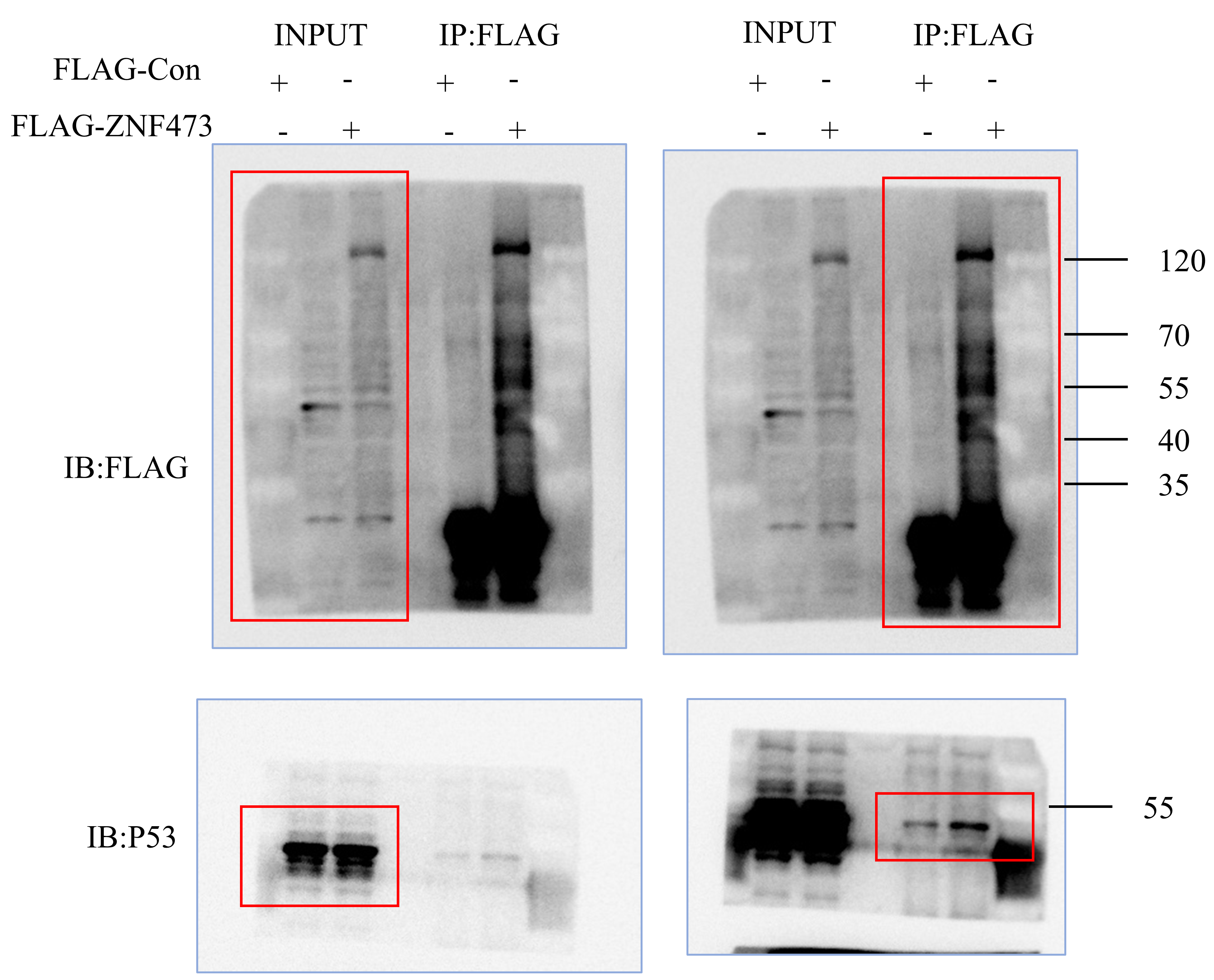
**

**Figure 5E**

**
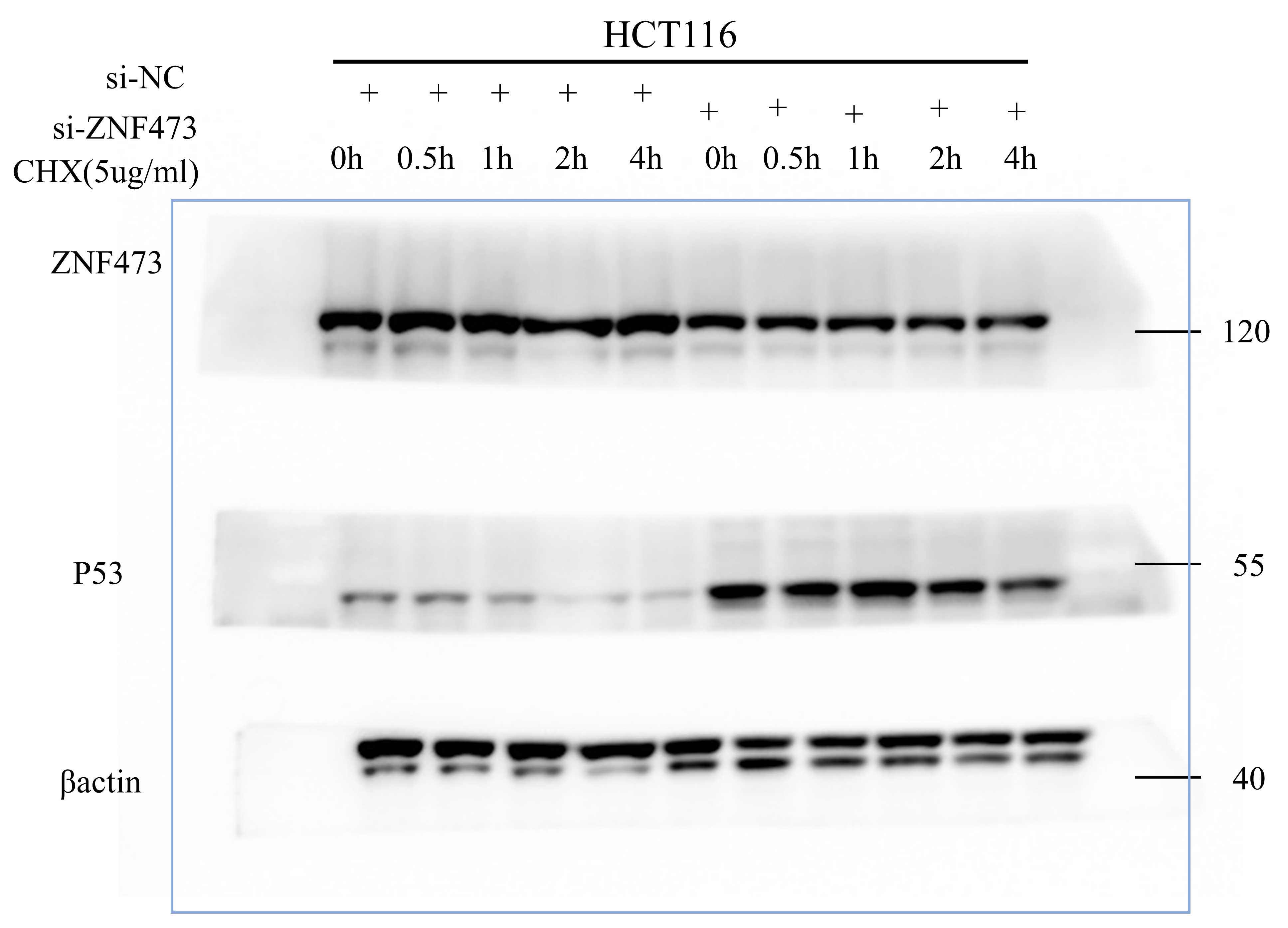
**

**Figure 6C**

**
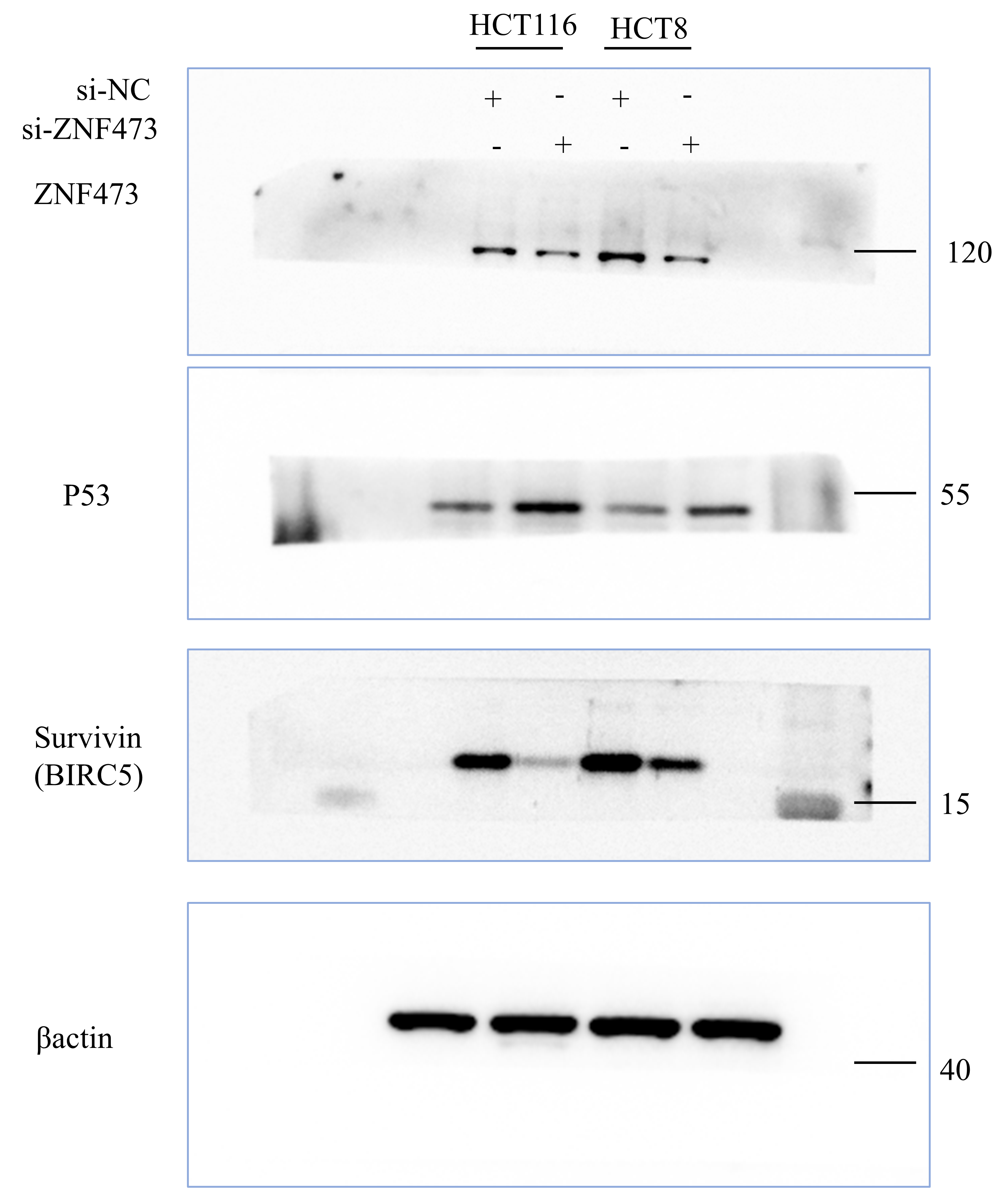
**

**Figure 6F-1**

**
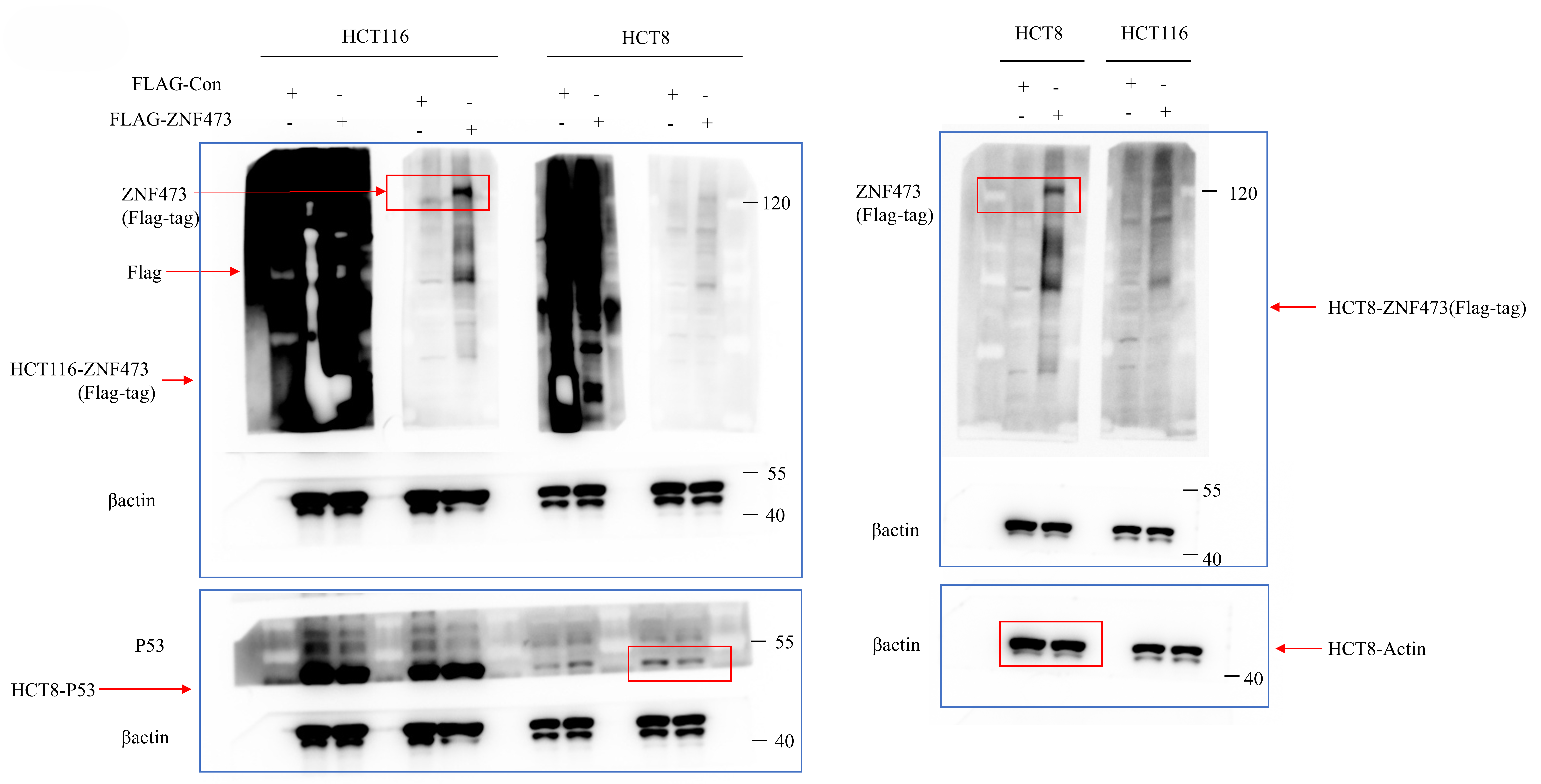
**

**Figure 6F-2**

**
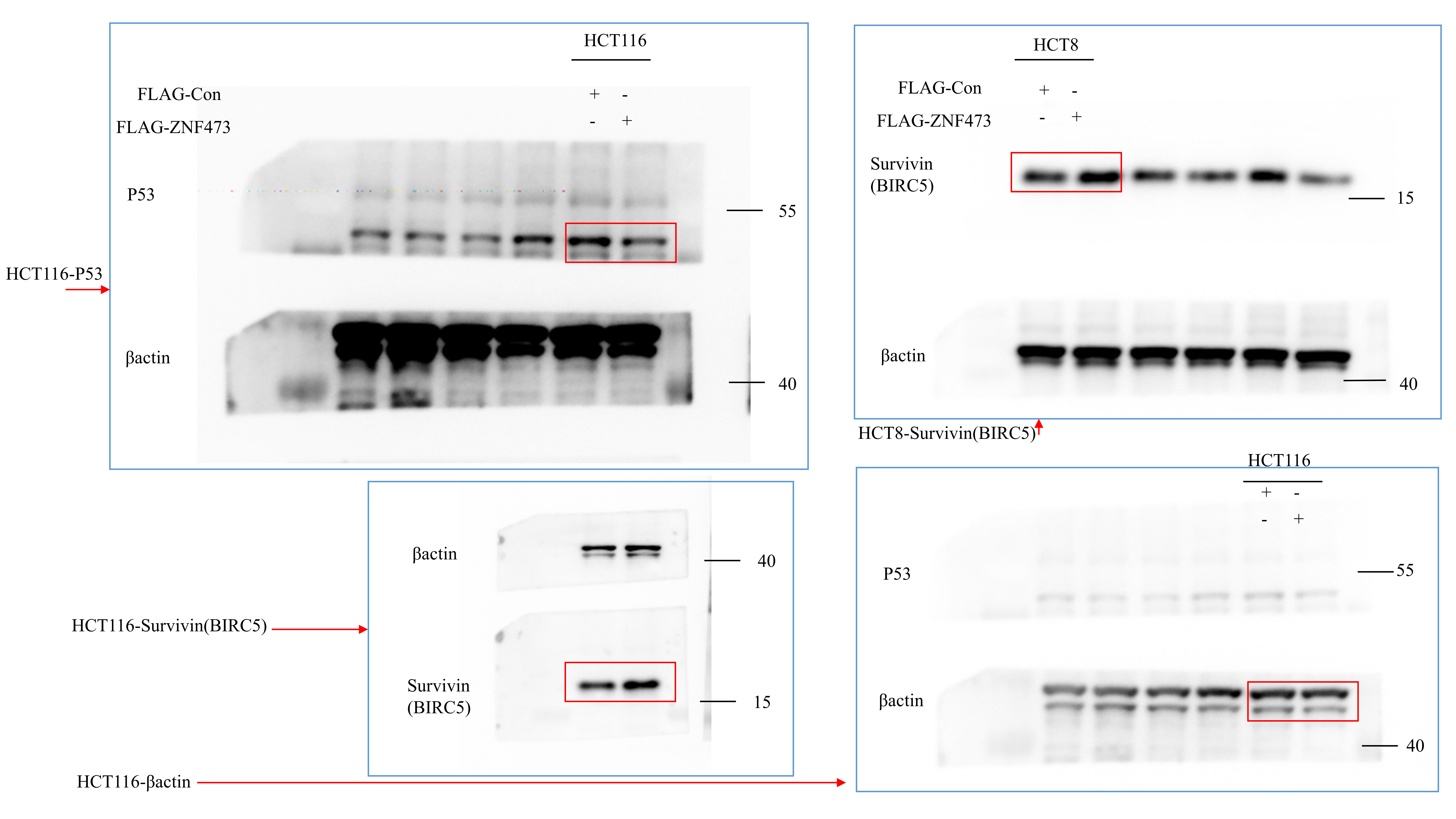
**

Supplement: Supplementary file 1 — Supplementary material [file 41420_2026_3145_MOESM1_ESM.docx]
